# Neuronal activity promotes secretory autophagy for the extracellular release of α-synuclein

**DOI:** 10.1016/j.jbc.2024.107419

**Published:** 2024-05-28

**Authors:** Yoshitsugu Nakamura, Taiki Sawai, Kensuke Kakiuchi, Shigeki Arawaka

**Affiliations:** Division of Neurology, Department of Internal Medicine IV, Osaka Medical and Pharmaceutical University Faculty of Medicine, Takatsuki, Osaka, Japan

**Keywords:** autophagy, glutamate, calcium, synuclein, Parkinson disease, protein secretion

## Abstract

Extracellular secretion is an essential mechanism for α-synuclein (α-syn) proteostasis. Although it has been reported that neuronal activity affects α-syn secretion, the underlying mechanisms remain unclear. Here, we investigated the autophagic processes that regulate the physiological release of α-syn in mouse primary cortical neurons and SH-SY5Y cells. Stimulating neuronal activity with glutamate or depolarization with high KCl enhanced α-syn secretion. This glutamate-induced α-syn secretion was blocked by a mixture of NMDA receptor antagonist AP5 and AMPA receptor antagonist NBQX, as well as by cytosolic Ca^2+^ chelator BAPTA-AM. Additionally, mTOR inhibitor rapamycin increased α-syn and p62/SQSTM1 (p62) secretion, and this effect of rapamycin was reduced in primary cortical neurons deficient in the autophagy regulator beclin 1 (derived from *BECN1*^+/−^ mice). Glutamate-induced α-syn and p62 secretion was suppressed by the knockdown of *ATG5*, which is required for autophagosome formation. Glutamate increased LC3-II generation and decreased intracellular p62 levels, and the increase in LC3-II levels was blocked by BAPTA-AM. Moreover, glutamate promoted co-localization of α-syn with LC3-positive puncta, but not with LAMP1-positive structures in the neuronal somas. Glutamate-induced α-syn and p62 secretion were also reduced by the knockdown of *RAB8A*, which is required for autophagosome fusion with the plasma membrane. Collectively, these findings suggest that stimulating neuronal activity mediates autophagic α-syn secretion in a cytosolic Ca^2+^-dependent manner, and autophagosomes may participate in autophagic secretion by functioning as α-syn carriers.

α-Synuclein (α-syn) plays a central role in the pathogenesis of Parkinson’s disease (PD), where it forms oligomers and insoluble aggregates that are toxic to neurons (1, 2). As shown by *GBA* gene variations, impairment of lysosomal degradation induces α-syn aggregation, indicating that protein homeostasis (proteostasis) of intracellular α-syn is key for preventing its toxic conversion and neurodegeneration in PD (3, 4). Elucidation of mechanisms that control the proteostasis of α-syn is important for understanding the processes underlying its toxic conversion. α-Syn is degraded by chaperone-mediated autophagy and macroautophagy (hereafter referred to as autophagy) (5, 6). The induction of autophagy and maturation of autophagosomes are thought to affect the proteostasis of α-syn *via* the degradative pathway under both basal and stress conditions (7). Additionally, autophagy has been shown to play a role in extracellular secretion of leaderless proteins that lack endoplasmic reticulum-targeting signal sequences (8, 9). As a leaderless protein, α-syn is also proposed to be secreted *via* autophagic processes in cell culture models (10, 11). This suggests that proteostasis of α-syn is maintained by a balance between the degradative and secretory pathways of autophagy. However, it is unclear how autophagic secretion of α-syn is regulated in neurons to maintain proteostasis.

Experiments using microdialysis have detected endogenous α-syn in the interstitial fluid of mice *in vivo*, indicating physiological α-syn secretion in healthy neurons (12). Additionally, neuronal activity induced by glutamate stimulates extracellular secretion of endogenous α-syn in primary cortical neurons, and neuronal activity evoked by the γ-aminobutyric acid A (GABA_A_) receptor antagonist picrotoxin stimulates α-syn secretion into the interstitial fluid of freely-moving mice (12). However, the mechanisms by which neuronal activity modulates α-syn secretion are not fully understood. In this study, we present that stimulating neuronal activity promotes autophagic secretion of α-syn and p62/SQSTM1 (hereafter referred to as p62) *via* cytosolic Ca^2+^ in mouse primary cortical neurons and SH-SY5Y cells.

## Results

### Neuronal activity mediates the physiological secretion of α-syn in a cytosolic Ca^2+^-dependent manner

**Figure 1:**
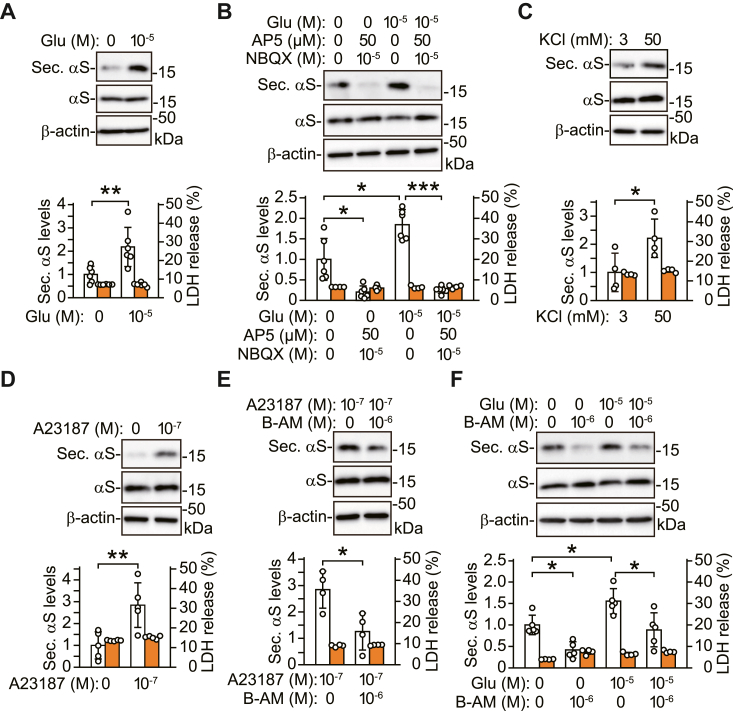
**Stimulating neuronal activity promotes physiological α-syn secretion in a cytosolic Ca**^**2+**^**-dependent manner in mouse primary cortical neurons.** TCA-precipitated CM and cell lysates are analyzed by Western blotting using antibodies against indicated proteins. β-actin is used as a loading control of cell lysates. *A*, treatment with 10 μM glutamate for 30 min increases α-syn secretion (n = 6). *B*, glutamate-induced α-syn secretion is blocked by adding a mixture of 50 μM AP5 and 10 μM NBQX (n = 6; *F*_(3,20)_ = 33.955, *p* < 0.001, ANOVA). LDH release is measured on each condition (n = 4). *C*, treatment with 50 mM KCl for 30 min increases α-syn secretion (n = 4), compared with 3 mM KCl. *D*, treatment with 100 nM A23187 for 6 h increases α-syn secretion (n = 5). *E*, A23187-induced α-syn secretion is blocked by adding 1 μM BAPTA-AM (n = 4). *F*, basal and glutamate-induced α-syn secretion is blocked by 1 μM BAPTA-AM (n = 5; *F*_(3,16)_ = 13.139, *p* < 0.001, ANOVA). LDH release is measured (n = 4). *A*–*F*, graphs show secreted α-syn levels normalized to controls (*white columns*) and percentages of LDH release to lysis buffer-treated positive controls (*orange columns*). Chemical treatments do not alter intracellular α-syn levels and extracellular release of LDH. Data represent mean ± SD. Data are analyzed by unpaired Student’s *t* test (*A*, *C*, *D*, and *E*), Welch’s ANOVA with Games-Howell’s *post hoc* tests (*B*), and one-way ANOVA with Bonferroni’s *post hoc* tests (*F*). ∗*p* < 0.05, ∗∗*p* < 0.01, ∗∗∗*p* < 0.001. B-AM, BAPTA-AM; Glu, glutamate; Sec. αS, secreted α-synuclein.

To elucidate how α-syn secretion is regulated, we examined whether neuronal activity affected α-syn secretion in mouse primary cortical neurons. In line with a previous study (12), treatment with glutamate enhanced endogenous α-syn secretion, compared with the basal condition (Fig. 1*A*). Glutamate-induced α-syn secretion was blocked by a mixture of N-methyl-d-aspartate (NMDA) receptor antagonist AP5 and AMPA receptor antagonist NBQX (Fig. 1*B*). Additionally, treatment with AP5 and NBQX suppressed basal α-syn secretion. These results suggest that stimulation and silencing of neuronal activity inversely affect α-syn secretion in neurons. Similar to the effect of glutamate, depolarization with highly concentrated KCl enhanced α-syn secretion (Fig. 1*C*). Next, we investigated whether neuronal activity affected α-syn secretion by changing cytosolic Ca^2+^ levels. Treatment with calcium ionophore A23187 increased α-syn secretion, and this effect of A23187 was inhibited by membrane-permeable Ca^2+^ chelator BAPTA-AM (Fig. 1, *D* and *E*). Basal and glutamate-induced α-syn secretion was also inhibited by BAPTA-AM (Fig. 1*F*). These treatments did not affect the intracellular levels of α-syn nor the extracellular release of LDH, indicating that they did not alter α-syn expression or neuronal viability. Together, these results suggest that stimulating neuronal activity enhances α-syn secretion in a cytosolic Ca^2+^-dependent manner, and that the basal α-syn secretion is also affected by free cytosolic Ca^2+^.

### Stimulating neuronal activity with glutamate enhances α-syn secretion *via* an autophagy-based mechanism

**Figure 2:**
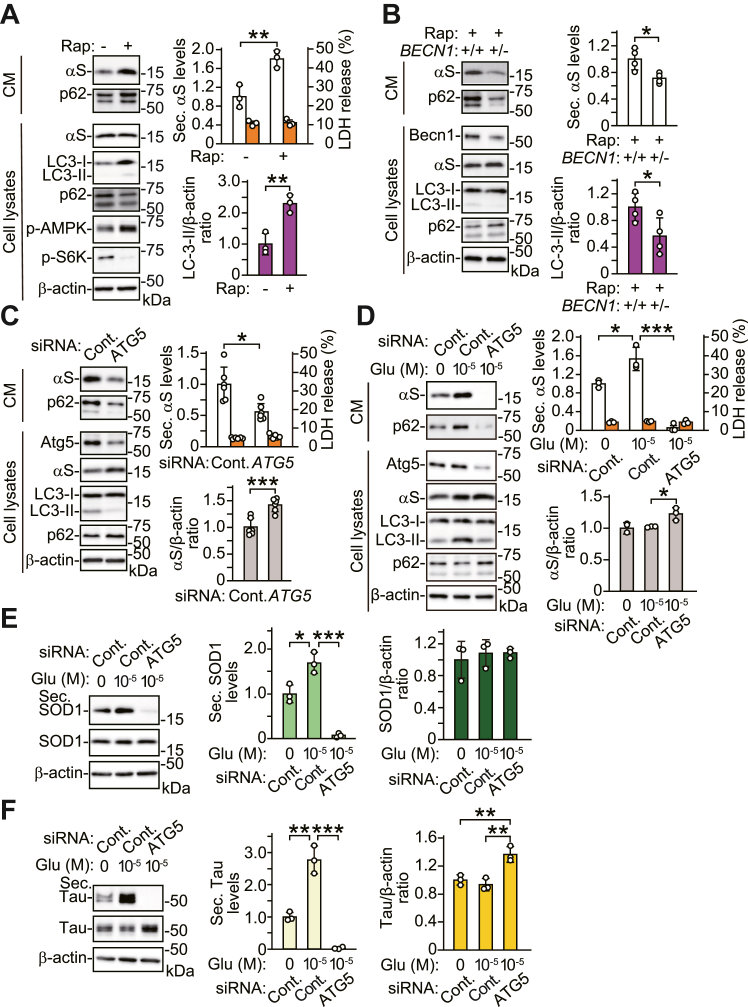
**Stimulating neuronal activity promotes α-syn secretion *via* an autophagy-based mechanism.** Western blots of TCA-precipitated CM and cell lysates with antibodies against indicated proteins are shown. *A*, treatment with 5 μM rapamycin for 6 h increases α-syn secretion and LC3-II generation in primary cortical neurons of wild-type littermate control mice (n = 3). *B*, rapamycin-induced α-syn secretion and LC3-II generation are suppressed in primary cortical neurons from *BECN1*^+/−^ mice, compared with those from wild-type littermates (n = 4). *C*, *ATG5* knockdown reduces α-syn secretion in wt-αS/SH cells (n = 6). *D*, glutamate-induced α-syn secretion is blocked by *ATG5* knockdown in wt-αS/SH cells (n = 3; *F*_(2,6)_ = 65.307, *p* < 0.001, ANOVA). *ATG5* knockdown increases intracellular α-syn levels in cells treated with glutamate (n = 3; *F*_(2,6)_ = 10.536, *p* < 0.05, ANOVA). *E*, glutamate-induced SOD1 secretion is blocked by *ATG5* knockdown in wt-αS/SH cells (n = 3; *F*_(2,6)_ = 62.218, *p* < 0.001, ANOVA). *F*, glutamate-induced tau secretion is blocked by *ATG5* knockdown in wt-αS/SH cells (n = 3; *F*_(2,6)_ = 84.046, *p* < 0.001, ANOVA). *ATG5* knockdown increases intracellular tau levels (n = 3; *F*_(2,6)_ = 19.251, *p* < 0.01, ANOVA). *Right upper graphs* of (*A*–*D*) show secreted α-syn levels normalized to controls (*white columns*) and percentages of LDH release to positive controls (*orange columns*). *Right lower graphs* of (*A* and *B*) show ratios of relative intensities (intracellular LC3-II to β-actin) normalized to controls (*purple columns*), and *right lower graphs* of (*C* and *D*) show ratios of relative intensities (intracellular α-syn to β-actin) normalized to controls (*gray columns*). *Left graphs* of (*E* and *F*) show secreted SOD1 (*light green columns*) and tau (*light yellow columns*) levels normalized to controls, respectively. *Right graphs* of (*E* and *F*) show ratios of relative intensities [intracellular SOD1 (*green columns*) and tau (*yellow columns*) to β-actin] normalized to controls, respectively. LDH release is unchanged in these experiments. Please note that secreted p62 levels of (*D*) are analyzed by Western blotting using anti-p62 antibody (#5114), and other data show the blots with anti-p62 antibody (#PM066). Data represent mean ± SD. Data are analyzed by unpaired Student’s *t* test (*A*–*C*) and one-way ANOVA with Bonferroni’s *post hoc* tests (*D*–*F*). ∗*p* < 0.05, ∗∗*p* < 0.01, ∗∗∗*p* < 0.001. Becn1, beclin 1; CM, conditioned media; Cont., control; Glu, glutamate; p-AMPK, phosphorylated AMPK; p-S6K, phosphorylated p70 S6 kinase; Rap, rapamycin; Sec. αS, secreted α-synuclein; Sec. p62, secreted p62; Sec. SOD1, secreted superoxide dismutase 1; Sec. Tau, secreted tau.

To evaluate the contribution of autophagy to α-syn secretion, we first characterized an antibody to autophagy cargo receptor protein p62, which is a key molecule in autophagic flux, in SH-SY5Y cells. In this study, we used an anti-p62 C-terminal antibody (#PM066) (13), which gives triplet signals on the Western blot (Fig. S1). A different anti-p62 antibody (#5114) recognized overexpressed FLAG-p62 and endogenous p62 protein. The endogenous p62 signal corresponded to the middle signal detected by the #PM066 antibody in SH-SY5Y cells. In this study, we indicated the middle signal as p62 on the blots with the #PM066 antibody. We then examined whether induction of autophagy altered α-syn secretion in primary cortical neurons. Treatment with mTORC1 inhibitor rapamycin stimulated autophagy (14), as shown by a reduction in p70 S6 kinase (p70S6K) phosphorylation and an increase in AMP-activated protein kinase (AMPK) phosphorylation (Fig. 2*A*). Rapamycin increased conversion from LC3-I to LC3-II, along with a reduction in intracellular p62 levels, indicating the enhancement of autophagic flux (Figs. 2*A* and S2*A*). Rapamycin concomitantly increased α-syn and p62 secretion, without affecting intracellular α-syn levels or LDH release. In contrast, rapamycin-mediated increases in α-syn and p62 secretion were significantly reduced in primary cortical neurons from *BECN1*-deficient (*BECN1*^+/−^) mice, compared with those from wild-type littermates (Fig. 2*B*). *BECN1* is the mammalian ortholog of the yeast autophagy-related (*ATG*) 6 gene, which is essential for the induction and regulation of autophagy (15). Intracellular beclin 1 levels were decreased to approximately 60% of those of wild-type littermates, and autophagic flux was suppressed, as shown by elevation of intracellular p62 levels (Figs. 2*B* and S2*B*). Intracellular α-syn levels were not affected (Fig. S2*B*). These findings suggest that α-syn is secreted *via* an autophagy-based mechanism. To assess whether neuronal activity affects autophagic α-syn secretion, we examined the effects of *ATG5*, which is required for autophagy induction (16), on α-syn secretion in a SH-SY5Y cell line stably expressing wild-type α-syn (wt-αS/SH) (17). siRNA-mediated knockdown of *ATG5* blocked LC3-II generation and increased intracellular p62 levels, indicating inhibition of autophagic flux (Figs. 2*C* and S2*C*). *ATG5* knockdown reduced α-syn and p62 secretion, compared with the non-silencing control, while it increased intracellular α-syn levels. Additionally, *ATG5* knockdown significantly suppressed glutamate-induced secretion of α-syn and p62, with an increase in intracellular α-syn levels, compared with the non-silencing control (Figs. 2*D* and S2*D*). LDH release was not altered. These results show that stimulating neuron-like activity with glutamate promotes autophagic processes involving α-syn and p62 secretion in SH-SY5Y cells.

To explore the mechanism for glutamate-induced autophagic secretion, we investigated whether neurodegeneration-related cytosolic proteins, superoxide dismutase 1 (SOD1) (18) and tau (19), were secreted *via* the autophagy-based mechanism. Treatment with glutamate increased secretion of SOD1 and tau proteins without altering their intracellular levels in wt-αS/SH cells (Fig. 2, *E* and *F*). The increases were significantly diminished by *ATG5* knockdown. *ATG5* knockdown increased intracellular tau levels, whereas it did not alter intracellular SOD1 levels. These findings suggest that stimulating neuron-like activity with glutamate promotes autophagic secretion of cytosolic proteins non-selectively in SH-SY5Y cells.

### Stimulating neuronal activity with glutamate affects autophagic flux in a cytosolic Ca^2+^-dependent manner

**Figure 3:**
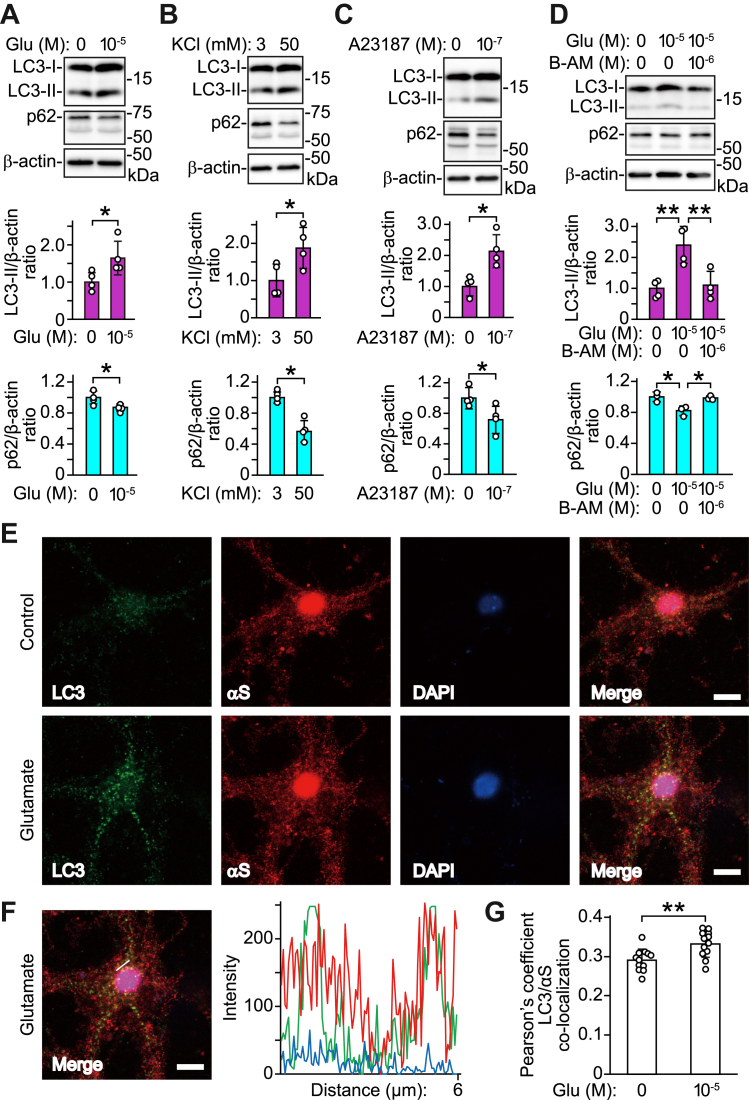
**Stimulating neuronal activity promotes autophagy and co-localization of α-syn and LC3-positive puncta in mouse primary cortical neurons.** Cell lysates are analyzed by Western blotting using antibodies against indicated proteins. Treatments with 10 μM glutamate for 30 min (*A*), 50 mM KCl for 30 min (*B*), and 100 nM A23187 for 6 h (*C*) increase the ratios of intracellular LC3-II levels to β-actin and decrease the ratios of intracellular p62 levels to β-actin (n = 4 in each group). *D*, glutamate-induced increase in the ratio of intracellular LC3-II levels and decrease in the ratio of intracellular p62 levels are inhibited by adding 1 μM BAPTA-AM (LC3-II: n = 4; *F*_(2,9)_ = 11.516, *p* = 0.003, ANOVA); (p62: n = 3; *F*_(2,6)_ = 10.445, *p* = 0.011, ANOVA). *E*, immunofluorescent analysis of primary cortical neurons treated with 10 μM glutamate for 30 min. Micrographs show images stained with anti-LC3 (4E12, *green*, *left panels*), anti-α-syn (ab6176, *red*, *left middle panel*s), and DAPI (*blue*, *right middle panels*). Right micrographs show merged images. Scale bar: 8 μm. *F*, co-localization line tracing analysis from images in (*E*). *G*, Pearson’s co-localization coefficient for LC3 and α-syn. Pearson’s coefficients were derived from three independent experiments with four fields per experiment, for a total of 12 fields contributing to the cumulative result. *A*–*D*, *upper* and *lower graphs* show ratios of relative intensities (intracellular LC3-II to β-actin) normalized to controls (*purple columns*) and ratios of relative intensities (intracellular p62 to β-actin) normalized to controls (*cyan columns*), respectively. Data represent mean ± SD. Data are analyzed by unpaired Student’s *t* test (*A*, *B*, *C*, and *G*) and one-way ANOVA with Bonferroni’s *post hoc* tests (*D*). ∗*p* < 0.05; ∗∗*p* < 0.01. αS, α-synuclein; B-AM, BAPTA-AM; Glu, glutamate.

**Figure 4:**
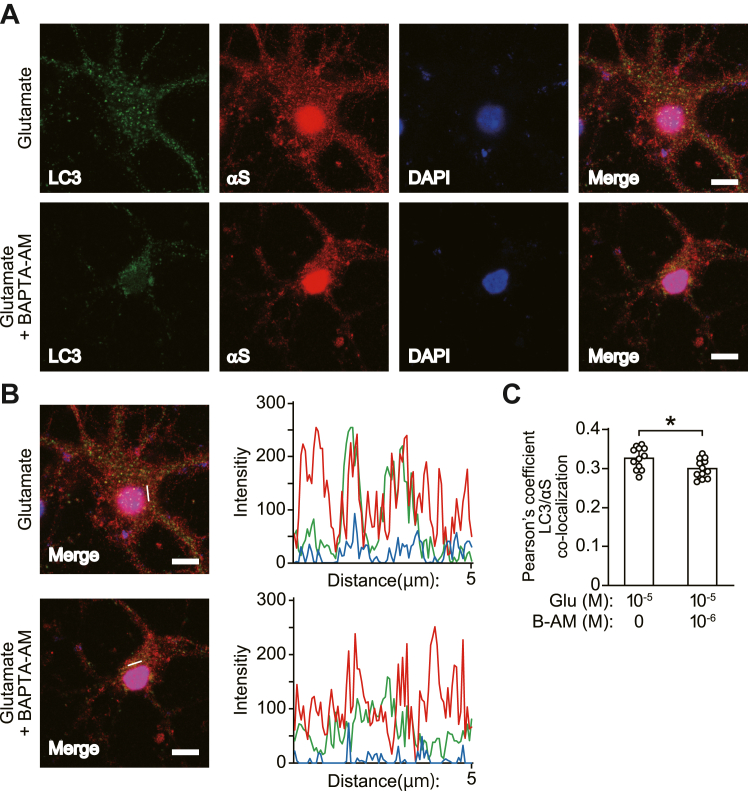
**Effect of BAPTA-AM on glutamate-induced co-localization of α-syn and LC3-positive puncta in mouse primary cortical neurons.** Immunofluorescent analysis of primary cortical neurons treated with 10 μM glutamate in the absence and presence of 1 μM BAPTA-AM for 30 min. *A*, micrographs show images stained with anti-LC3 (4E12, *green*, *left panels*), anti-α-syn (ab6176, *red*, *left middle panels*), and DAPI (*blue*, *right middle panels*). Right micrographs show merged images. Scale bar: 8 μm. *B*, co-localization line tracing analysis from images in (*A*). *C*, Pearson’s co-localization coefficient for LC3 and α-syn. Pearson’s coefficients were derived from three independent experiments with four fields per experiment, for a total of 12 fields contributing to the cumulative result. Data represent mean ± SD. Data are analyzed by unpaired Student’s *t* test (*C*). ∗*p* < 0.05. αS, α-synuclein; B-AM, BAPTA-AM; Glu, glutamate.

To explore a functional link between neuronal activity and autophagic secretion of α-syn, we assessed whether neuronal and synaptic activities affected autophagic flux in primary cortical neurons. Treatment with glutamate or KCl increased intracellular LC3-II levels and decreased intracellular p62 levels (Fig. 3, *A* and *B*). A23187 treatment similarly facilitated conversion to LC3-II, while reducing intracellular p62 levels (Fig. 3*C*). The glutamate-induced increase in the LC3-II levels was blocked by BAPTA-AM (Fig. 3*D*). In line with the findings of Western blot analysis, immunofluorescence analysis demonstrated that glutamate treatment promoted co-localization of α-syn and puncta that stained positively for LC3 (a marker of autophagosomes) in the neuronal somas (20) (Fig. 3, *E*–*G*). When primary cortical neurons were treated with BAPTA-AM, glutamate-induced co-localization of α-syn and LC3 puncta was abrogated (Fig. 4). These findings show that stimulating neuronal activity with glutamate enhances autophagic flux in a cytosolic Ca^2+^-dependent manner and promotes transport of α-syn to autophagosomes.

### Stimulating neuronal activity with glutamate affects α-syn secretion *via* autophagosomes

**Figure 5:**
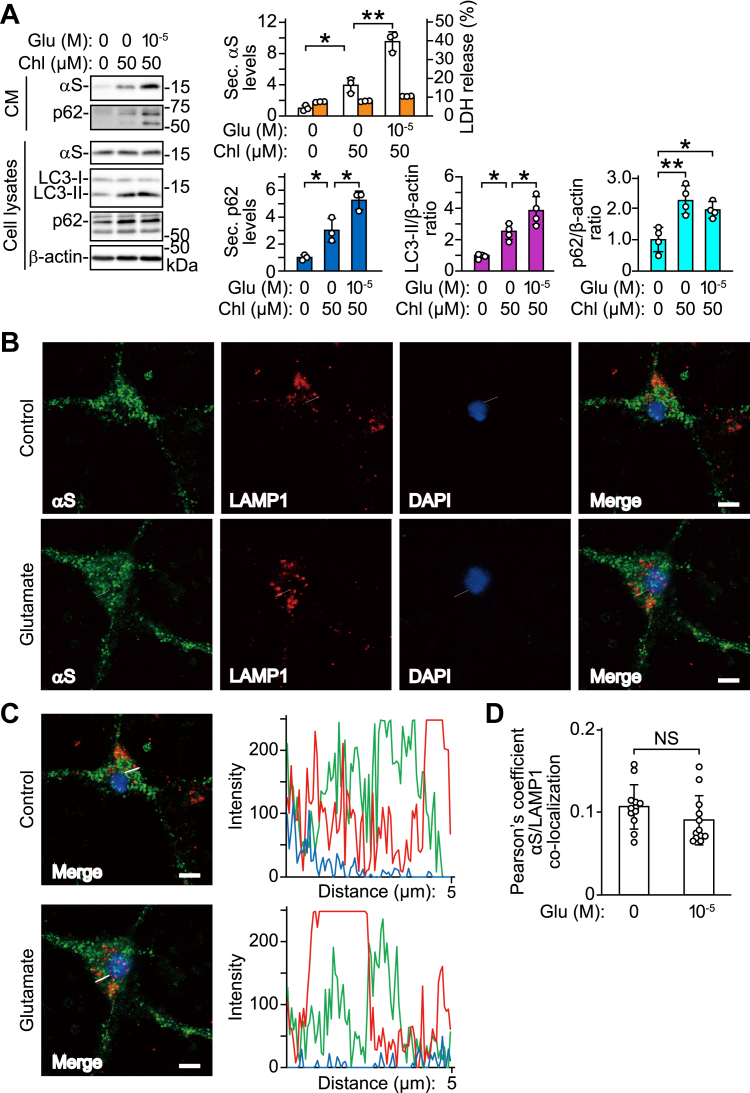
**Effects of neuronal activity on autophagic processes involving α-syn secretion in mouse primary cortical neurons.***A*, primary cortical neurons were pre-treated with 50 μM chloroquine for 6 h, followed by co-treatment with 10 μM glutamate for 30 min. TCA-precipitated CM and cell lysates are analyzed by Western blotting using antibodies against indicated proteins. Glutamate treatment increases α-syn and p62 secretion and LC3-II generation in the presence of chloroquine, compared with chloroquine alone (secreted α-syn: n = 3; *F*_(2,6)_ = 65.205, *p* < 0.001, ANOVA); (secreted p62 secretion: n = 3; *F*_(2,6)_ = 31.320, *p* = 0.001, ANOVA); (intracellular LC3-II: n = 4; *F*_(2,9)_ = 22.945, *p* < 0.001, ANOVA); (intracellular p62: n = 4; *F*_(2,9)_ = 11.994, *p* = 0.003, ANOVA). *B*, immunofluorescent analysis of primary cortical neurons treated with 10 μM glutamate for 30 min. Micrographs show images stained with anti-α-syn (42/α-Synuclein, *green*, *left panels*), anti-LAMP1 (EPR21026, *red*, *left middle panels*), and DAPI (*blue*, *right middle panels*). Right micrographs show merged images. Scale bar: 8 μm. *C*, co-localization line tracing analysis from images of cells in (*B*). *D*, Pearson’s co-localization coefficient for α-syn and LAMP1. Pearson’s coefficients were derived from three independent experiments with four fields per experiment, for a total of 12 fields contributing to the cumulative result. Upper graph of (*A*) shows secreted α-syn levels normalized to controls (*white columns*) and percentages of LDH release to positive controls (*orange columns*). Lower graphs show secreted p62 levels normalized to controls (*left*, *blue columns*), ratios of relative intensities (intracellular LC3-II to β-actin) normalized to controls (*middle*, *purple columns*) and ratios of relative intensities (intracellular p62 to β-actin) normalized to controls (*right*, *cyan columns*). Data represent mean ± SD. Data are analyzed by one-way ANOVA with Bonferroni’s *post hoc* tests (*A*) and unpaired Student’s *t* test (*D*). ∗*p* < 0.05; ∗∗*p* < 0.01. Chl, chloroquine; CM, conditioned media; Glu, glutamate; NS, not significant; Sec. αS, secreted α-synuclein; Sec. p62, secreted p62.

Chloroquine blocks the fusion of autophagosomes with lysosomes and inhibits autophagic degradation (21, 22). Treatment with chloroquine led to an increase in GFP-LC3 puncta and co-localization of GFP-LC3 puncta and p62 in AcGFP1-LC3 cDNA-transfected SH-SY5Y cells (Fig. S3). To investigate the autophagic processes underlying neuronal activity-mediated α-syn secretion, we treated primary cortical neurons with chloroquine. Treatment with chloroquine increased α-syn and p62 secretion, and co-treatment with glutamate further promoted α-syn and p62 secretion, compared with a single treatment with chloroquine (Fig. 5*A*). These treatments did not alter intracellular α-syn levels and LDH release. Treatment with chloroquine increased intracellular levels of p62 and LC3-II. Co-treatment with glutamate further increased intracellular levels of LC3-II, whereas those of p62 were unchanged. These results suggest that stimulation of neuronal activity with glutamate promotes autophagosome formation in cells subjected to chloroquine-induced lysosome inhibition. Additionally, immunofluorescence analysis showed that treatment with glutamate did not promote co-localization of α-syn and structures that stained positively for LAMP1 (a marker of late endosomes and lysosomes) in primary cortical neurons (23) (Fig. 5, *B*–*D*).

**Figure 6:**
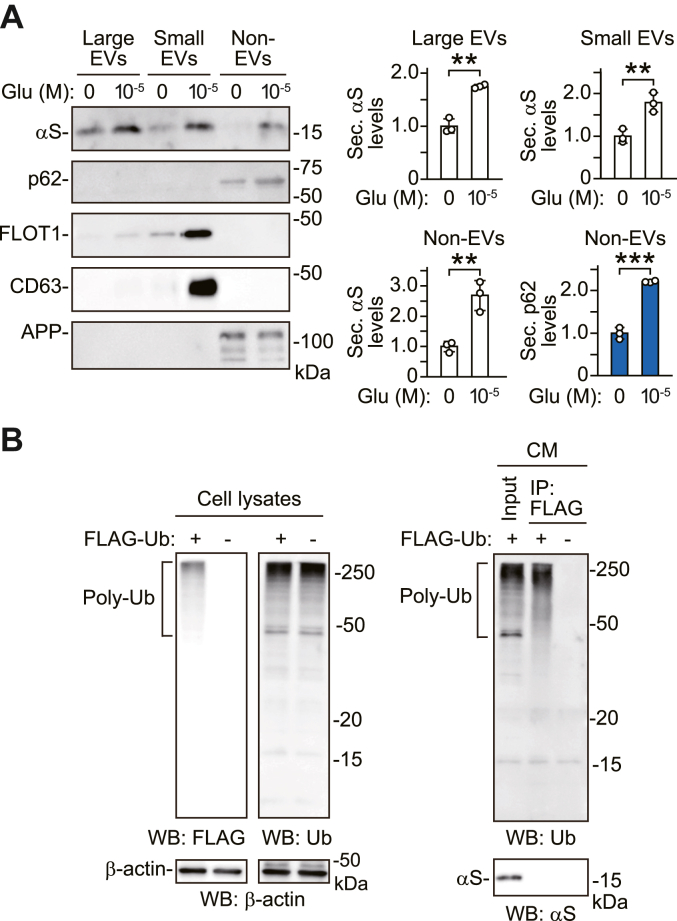
**Assessment of secreted α-syn forms under basal and neuronal activity-stimulating conditions.***A*, CM of primary cortical neurons treated with 10 μM glutamate for 30 min were fractionated by step-wise centrifugation and separated into large EV, small EV and non-EV fractions. Each fraction is analyzed by Western blotting using antibodies against indicated proteins. *Right graph*s show secreted α-syn levels normalized to controls in each fraction (*white columns*) and secreted p62 levels normalized to controls in non-EV fractions (*blue column*s) (n = 3). APP is used as a marker secreted in free-protein forms. *B*, immunoprecipitation (IP) analysis of ubiquitinated proteins in CM of wt-αS/SH cells. TCA-precipitated CM were dissolved with RIPA buffer and subjected to IP with anti-FLAG antibody. *Left panels* show Western blots of cell lysates using indicated antibodies. Anti-FLAG antibody detects the smear of poly-ubiquitin (Ub) conjugates only in cells transfected with FLAG-tagged Ub cDNA. The signals are matched with poly-Ub conjugates visualized by anti-Ub antibody. *Right panels* show the blots of the input material and IP products. In the IP products from cells transfected with FLAG-tagged Ub cDNA, the blots with anti-Ub antibody detect poly-Ub conjugate signals. However, there is no signal of α-syn in the blots of the IP products with anti-α-syn antibody. Data represent mean ± SD. Data are analyzed by unpaired Student’s *t* test (*A*). ∗∗*p* < 0.01, ∗∗∗*p* < 0.001. αS, α-synuclein; APP, amyloid-β precursor protein; CM, conditioned media; EV, extracellular vesicle; FLOT1, flottilin1; Glu, glutamate; IP, immunoprecipitation; Sec. αS, secreted α-synuclein; Sec. p62, secreted p62; Ub, ubiquitin; WB, Western blot.

To investigate secretory forms of α-syn under stimulating neuronal activity with glutamate, we fractionated conditioned media (CM) of primary cortical neurons by step-wise centrifugation in the absence and presence of glutamate (Fig. 6*A*). In this fractionation, post-10,000*g* pellets, post-110,000*g* pellets, and post-110,000*g* supernatants were collected as large extracellular vesicle (EV), small EV, and non-EV fractions (24). Treatment with glutamate increased the amounts of secreted α-syn with exosome markers, flotillin-1 and CD63, in small EV fractions (25). Glutamate also increased the amounts of secreted α-syn in non-EV fractions, although their signals were faint. These data showed that stimulating neuronal activity with glutamate extracellularly released α-syn in the exosome-associated and free-protein forms. Glutamate-induced α-syn secretion was also seen in large EV fractions, suggesting that α-syn is secreted in multiple forms under stimulation of neuronal activity with glutamate. Autophagic cargo receptor p62 was demonstrated to be secreted in small EV fractions with the binding to the cytoplasmic side of exosome membranes (24). Although the exact reason was unclear, the present data showed that basal and glutamate-induced secretion of p62 occurred in non-EV fractions. Previous study has demonstrated that ubiquitinated α-syn is transported into lysosomes (26). To assess whether secreted α-syn is ubiquitinated, we performed immunoprecipitation of CM in SH-SY5Y cells transfected with FLAG-tagged ubiquitin (Ub) or empty vector cDNA. Anti-FLAG antibody specifically detected poly-ubiquitin (Ub) conjugate signals on the blots of lysates prepared from cells transfected with FLAG-tagged Ub cDNA (Fig. 6*B*). Anti-Ub antibody recognized poly-Ub conjugates on the blots of lysates from cells transfected with FLAG-tagged Ub or empty vector cDNA. When radioimmunoprecipitation assay (RIPA) lysates from cells transfected with FLAG-tagged Ub cDNA were immunoprecipitated with anti-FLAG antibody, we detected poly-Ub conjugate signals on the blots with anti-Ub antibody. However, in the immunoprecipitated products, there were no α-syn signals on the blots with anti-α-syn antibody, although α-syn signals were found in the input material on the blots with anti-α-syn antibody. When RIPA lysates from cells transfected with empty vector cDNA were immunoprecipitated with the anti-FLAG antibody, there were no poly-Ub conjugate signals on the blots with anti-Ub antibody. Immunoprecipitation assay showed that secreted α-syn was not ubiquitinated in SH-SY5Y cells. Collectively, these results support that autophagosomes before fusion with lysosomes play a role in neuronal activity-mediated secretion of α-syn.

### Effects of Rab7 and Rab8a on autophagosomal secretion of α-syn

**Figure 7:**
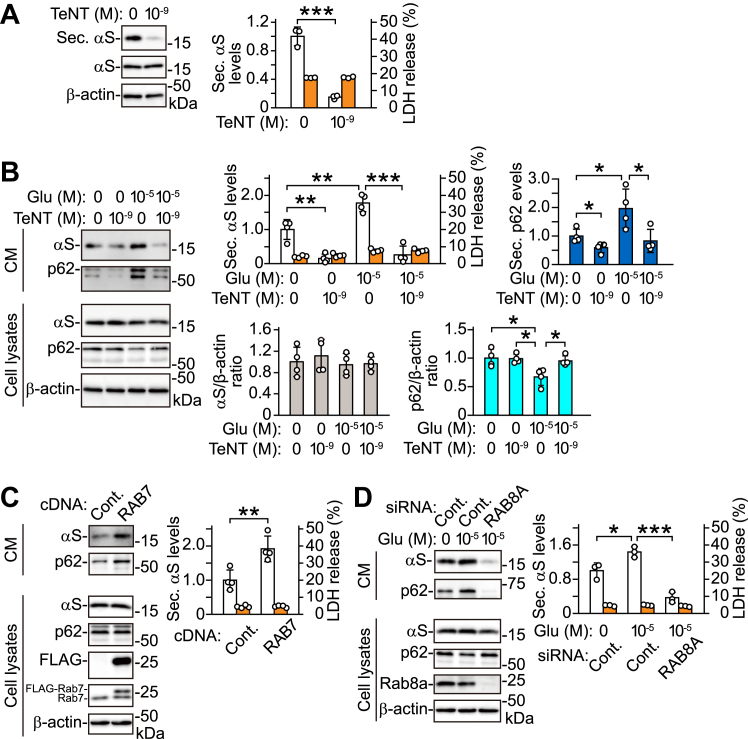
**Role of autophagosomes in neuronal activity-mediated α-syn secretion.** Western blots of TCA-precipitated CM and cell lysates are shown. *A*, treatment with 1 nM TeNT for 24 h inhibits α-syn secretion in primary cortical neurons (n = 3). *B*, glutamate-induced α-syn and p62 secretion is blocked by adding 1 nM TeNT in primary cortical neurons (secreted α-syn: n = 4; *F*_(3,12)_ = 39.812, *p* < 0.001, ANOVA); (secreted p62: n = 4; *F*_(3,12)_ = 8.010, *p* = 0.003, ANOVA); (intracellular α-syn: n = 4; *F*_(3,12)_ = 0.393, *p* = 0.76, ANOVA) (intracellular p62: n = 4; *F*_(3,12)_ = 6.479, *p* = 0.007, ANOVA). *C*, overexpression of Rab7 increases α-syn secretion in wt-αS/SH cells (n = 4). *D*, glutamate-induced α-syn secretion is blocked by *RAB8A* knockdown in wt-αS/SH cells (n = 3; F_(2,6)_ = 40.114, *p* < 0.001, ANOVA). *A*–*D*, graphs show secreted α-syn levels normalized to controls (*white columns*) and percentages of LDH release to positive controls (*orange columns*). *Upper right graph* of (*B*) shows secreted p62 levels normalized to controls (*blue columns*). *Lower left* and *right graphs* of (*B*) show ratios of relative intensities (intracellular α-syn to β-actin) normalized to controls (*gray columns*) and ratios of relative intensities (intracellular p62 to β-actin) normalized to controls (*cyan columns*), respectively. Intracellular α-syn levels and LDH release are unchanged in these experiments. Data represent mean ± SD. Data are analyzed by unpaired Student’s *t* test (*A* and *C*) and one-way ANOVA with Bonferroni’s *post hoc* tests (*B* and *D*). ∗*p* < 0.05, ∗∗*p* < 0.01, ∗∗∗*p* < 0.001. CM, conditioned media; Cont., control; Glu, glutamate; Sec. αS, secreted α-synuclein; Sec. p62, secreted p62; TeNT, tetanus toxin.

We investigated whether SNARE (soluble N-ethylmaleimide-sensitive factor attachment protein receptor) proteins played a role in the autophagic secretion of α-syn. Tetanus toxin (TeNT) is known to decrease the size of Atg16L1 vesicles, possibly affecting autophagosome formation *via* inhibition of the SNARE members VAMP1, VAMP2 and VAMP3 (27). Treatment with TeNT reduced basal secretion of α-syn and p62, without altering their intracellular levels or LDH release in primary cortical neurons (Fig. 7, *A* and *B*). TeNT also reduced the glutamate-induced secretion of α-syn and p62, while restoring intracellular p62 to basal levels (Fig. 7*B*). These findings suggest that neuronal activity-mediated autophagic secretion is affected by an early step in autophagosome formation. We investigated the effects of a sub-family of Rab GTPases on the autophagic secretion of α-syn. Rab7 (herein, referring to the mammalian Rab7a isoform, a homolog of yeast Ypt7) plays a role in maturation of the endosome–lysosome system and in fusion of autophagosomes with late endosomes and lysosomes (28, 29). Overexpression of Rab7 enhanced the basal secretion of α-syn and p62 in SH-SY5Y cells, suggesting that basal autophagic secretion of α-syn might be affected by promoting autophagosome maturation (Fig. 7*C*). Rab8a is a regulator of polarized membrane trafficking and fusion of autophagosomes and amphisomes to the plasma membrane (PM) (8, 30). siRNA-mediated knockdown of *RAB8A* significantly suppressed glutamate-induced secretion of α-syn and p62 in SH-SY5Y cells (Fig. 7*D*). *RAB8A* knockdown did not affect intracellular α-syn levels or LDH release. *RAB8A* knockdown blocked the glutamate-induced reduction in intracellular p62. These findings suggest that neuron-like activity-mediated autophagic secretion of α-syn and p62 is affected by Rab8a-associated fusion of autophagosomes to the PM in SH-SY5Y cells.

## Discussion

The mechanisms regulating neuronal autophagy are a major focus of studies on the physiological and pathological roles of autophagy. Treatment with low-dose NMDA, which induces chemical long-term depression, rapidly increases autophagic flux and the number of GFP-LC3 puncta in dendrites and spines of hippocampal neurons *via* receptor activation (31). These data indicate that stimulation of neuronal activity triggers autophagy. In addition to degradation, autophagy functions as a secretory carrier (9). However, whether secretory autophagy is triggered by stimulating neuronal activity is largely unexplored. Here, we show that stimulating neuronal activity promotes autophagic flux for secretion, which mediates the physiological release of α-syn.

We found that blocking glutamate receptors by AP5 and NBQX and chelating cytosolic Ca^2+^ by BAPTA-AM decreased basal and glutamate-induced α-syn secretion. These findings suggest that Ca^2+^ influx by activation of glutamate receptors mediates neuronal α-syn secretion. The previous study has also shown that treatment with α-latrotoxin (αLTX) promotes α-syn secretion, and the promoting effect of αLTX is abolished by adding the extracellular Ca^2+^ chelator EGTA in primary neurons (12). These data are interpreted by a scenario that synaptic vesicle exocytosis induces α-syn secretion because αLTX enhances synaptic vesicle release *via* Ca^2+^ influx (12). We cannot exclude this interpretation because the synaptic vesicle exocytosis blocker TeNT (32) dampened basal and glutamate-induced α-syn secretion. However, we further found that neuronal activity-mediated α-syn secretion was decreased by knockdowns of *ATG5* and *RAB8A* that modulate autophagosome formation (16) and autophagosomal secretion (8, 30), respectively. Elevation in cytosolic Ca^2+^ *per se* may trigger autophagic secretion of α-syn in neurons. Ca^2+^ acts as a positive or negative regulator, depending the step of autophagic processes (33). How Ca^2+^ influx by prolonged stimulation of glutamate receptors affects steps of autophagic secretion remains questions to be answered.

The present results showed that stimulating neuronal activity with glutamate did not affect the intracellular α-syn levels. This suggests that the increase in α-syn secretion is insufficient to reduce its intracellular levels. Alternatively, stimulating neuronal activity might not increase the autophagic degradation of α-syn. Unexpectedly, stimulating neuronal activity with glutamate did not promote co-localization of α-syn and LAMP1-positive structures, although it induced co-localization of α-syn and LC3-positive puncta in the neuronal somas. These results suggest that stimulating neuronal activity promotes sequestration and packaging of α-syn into autophagosomes, but not its trafficking into autolysosomes for degradation. In support of this hypothesis, stimulating neuronal activity with glutamate increased LC3-II generation without affecting intracellular p62 levels under chloroquine-induced inhibition of autophagosome–lysosome fusion. Additionally, the neuronal activity-mediated α-syn secretion was dependent on Rab8a. Rab8a is required for autophagic secretion of IL-1β under conditions of starvation and inflammasome activation (8). Rab8a is also demonstrated to control TGFB1 release *via* secretory autophagosomes (30). Collectively, these findings suggest that stimulating neuronal activity may promote the steps of autophagosome formation prior to fusion with lysosomes, and that autophagosomes may play a key role in neuronal activity-mediated secretory autophagy.

How α-syn is captured by autophagic vacuoles? The present data showed that stimulating neuronal activity with glutamate pushed secretion of α-syn collected in small EV fractions, which abundantly contain exosomes, in primary cortical neurons. This finding suggests that considerable amounts of secreted α-syn originate from endosomes, and the fusion of autophagosomes with endosomes is a key event for this autophagic secretion of α-syn. Many studies have described that α-syn is associated with exosomes (11, 22, 34, 35). However, it should be noted that the step-wise fractionation does not clarify whether α-syn is packaged within the lumen side of exosomes or it attaches to the cytoplasmic side of exosomes, as shown in p62 (24). Unexpectedly, we found that treatment with glutamate enhanced α-syn secretion in large EV fractions, indicating that α-syn is extracellularly released by different membranous vesicles, such as microvesicles (11). In this study, we did not perform electron microscopic analysis for various vesicles. It is necessary to identify membranous vesicles that transport α-syn into the extracellular space and to assess how these transport systems of membranous vesicles depend on autophagy. As a different route, we found that treatment with glutamate enhanced the secretion of α-syn in non-EV fractions. Additionally, ubiquitinated α-syn was undetectable in CM under basal conditions. These findings raised a possibility that autophagic vacuoles captured α-syn in a non-selective manner. Although the exact mechanism remains unclear, treatment with glutamate enhanced the secretion of cytosolic proteins including SOD1 and tau. Accumulation of misfolded SOD1 and tau proteins are considered to be causatively associated with the pathogenesis of hereditary amyotrophic lateral sclerosis and tauopathies, respectively (18, 19). Neuronal activity-mediated secretion may relate to non-selective bulk autophagy to extracellularly release cytoplasmic proteins and contribute to proteostasis of these molecules associated with neurodegenerative diseases.

Although it is unclear to what extent stimulating neuronal activity affects autophagic degradation and whether there are molecular requirements in the recruitment of cargos into autophagosomes for secretion, functional switching between degradation and secretion may occur for autophagic regulation of proteostasis in cells (9). In the fly neuromuscular junction, neuronal activity with high K^+^ has been demonstrated to block autophagosome-lysosome fusion, whereas it drives the pathway toward autophagy-based secretion (36). A previous study has shown that syntaxin 17 promotes autophagosome-lysosome fusion and cargo degradation, and Sec22b promotes autophagic secretion of IL-1β by fusion of autophagosomes to the PM (37). The difference in the SNARE machinery coating the cytosolic membrane of autophagosome is proposed to control the functional switching between autophagic degradation and secretion (38). Stimulation of synaptic activity with 4-aminopyridine and bicuculline, an antagonist of voltage-gated potassium channels and inhibitory GABA_A_ receptors, respectively, has been shown to decrease autophagic vacuole motility in dendrites, but not in axons, while increasing degradative autolysosomes in dendrites (39). The molecular mechanisms underlying the functional switching of autophagosomes towards the secretory or degradative pathway in the various neuronal compartments need to be clarified in future studies. The impact of autophagy on α-syn pathology has generally been investigated from the perspective of dysfunctional degradation (5, 40). However, little is unknown about how secretory autophagy contributes to the intracellular clearance of α-syn or whether impairment of secretory autophagy modulates the abnormal accumulation and spread of α-syn pathology. Further studies are warranted to elucidate the mechanisms controlling the secretion of α-syn oligomers in neurons and glial cells, as well as to uncover the role of dysregulated α-syn proteostasis in disease models.

## Experimental procedures

### Materials

Reagents for cell culture experiments and chemical reagents were obtained from Thermo Fisher Scientific and Sigma-Aldrich, respectively, unless otherwise stated.

### Cell culture, siRNA transfection, and LDH assay

Primary cortical neuron cultures were obtained from ICR mice (Japan SLC, Inc) (41). Also, primary cortical neurons were obtained from *BECN1*-deficient mice and wild-type littermates (C57B6/J genetic background, #028796, Jackson laboratory) (42). We used heterozygous deficient mice because *BECN1*-homozygous deficiency is neonatal lethal. Neurons were isolated from the neocortex of embryonic day 17 mice and dissociated cells were plated at a density of 1.5 × 10^6^ cells on poly-L-lysine (PLL, Sigma-Aldrich)-coated 6-well plates. Neurons were maintained in serum-free neurobasal plus medium supplemented with B-27 plus supplement, GlutaMAX and 0.05% 2-mercapthoehanol (2-ME) at 37 °C in 5% CO_2_ atmosphere. At intervals of 3 to 4 days, half of the plating medium was renewed. At 11 to 12 days *in vitro*, neurons were used for experiments. Protocols were approved by the Animal Subjects Committees of Osaka Medical and Pharmaceutical University (#AM23-005 and #AM23-007). The human dopaminergic neuroblastoma SH-SY5Y cell line stably expressing wild-type α-syn (#ECACC 94030304, wt-αS/SH) (17) was maintained in Eagle’s minimum essential medium/Ham’s F-12 (Sigma-Aldrich) supplemented with 15% fetal bovine serum, 2 mM L-glutamine and 1× non-essential amino acids (Sigma-Aldrich) and used for siRNA knockdown and cDNA overexpression.

We transiently transfected 5 μg of human cDNAs with a FLAG tag at the C terminus subcloned into the pcDNA3.1 vector (Genscript) or AcGFP1-LC3 subcloned into the pAutophagSENSE Vector (Takara) with 5 × 10^6^ cells using the Amaxa Nucleofector device (Lonza). At 48 h after transfection, the cells were used for experiments. For siRNA-mediated knockdown, approximately 20% confluent wt-αS/SH cells were transfected with 10 nM siRNA oligonucleotides (Silencer Select RNAi) using RNAiMAX reagent (Thermo Fisher Scientific) (43). The sequences are as follows: human *ATG5*, 5′-GGAUGCAAUUGAAGCUCAUtt-3′ (Thermo Fisher Scientific, s18158); *RAB8A*, GUCAAAAUCACACCGGAtt-3′ (s8680). As a non-silencing control, Silencer Select Negative Control #1 siRNA (Thermo Fisher Scientific) was used. At 72 h after siRNA transfection, the cells were re-transfected and incubated for 48 h.

To assess cell membrane damage, LDH assay (Dojindo) was performed under the same conditions as corresponding experiments. 10% lysis buffer was added to the media to induce maximum LDH release as a positive control. LDH release was calculated as a percentage relative to a positive control.

### Chemical treatments

Neurons and cells were treated with 10 μM glutamate or 50 mM KCl for 30 min. Neurons were pre-treated with 50 μM AP5 (Abcam) and 10 μM NBQX (Abcam) for 2 h and then treated with a mixture of glutamate, AP5, and NBQX for 30 min. Neurons were also treated with 100 nM A23187 for 6 h. To see the effects of cytosolic Ca^2+^, neurons were pre-treated with 1 μM BAPTA-AM (Toronto Research Chemicals) for 2 h and then co-treated with 10 μM glutamate for 30 min or 100 nM A23187 for 1 h. Neurons were incubated in media containing 5 μM rapamycin (Fujifilm) for 6 h. For chloroquine treatment, neurons were incubated in media containing 50 μM chloroquine for 6 h. To see the effects of TeNT on α-syn secretion, neurons were treated with 1 nM TeNT for 24 h. In chemical treatments, neurons and cells were treated with the equivalent volume of the vehicle as a control.

### Preparation of cell lysates and CM

To obtain cell lysates, neurons and cells were suspended in ice cold lysis buffer [20 mM Tris-HCl, pH 7.4, 150 mM NaCl, 1% Triton X-100, 10% glycerol, 1× protease inhibitor cocktail (Roche Diagnostic), 1 mM EDTA, 5 mM NaF, 1 mM Na_3_VO_4_, 1× phosSTOP (Roche Diagnostic)], sonicated at 30 W for 3 s five times, and kept on ice for 30 min (41). Samples were centrifugated at 12,000*g* for 30 min at 4 °C. The resultant supernatants were collected as cell lysates and stored at −80 °C until use. Protein concentration was measured by the BCA Protein Assay Kit (Takara).

To obtain CM in treatment with glutamate or KCl, neurons, and cells were replaced from growth media to fresh artificial cerebrospinal fluid (1.3 mM CaCl_2_, 1.2 mM MgSO_4_, 3 mM KCl, 0.4 mM KH_2_PO_4_, 25 mM NaHCO_3_, and 122 mM NaCl, pH 7.4). In other experiments, primary cortical neurons and SH-SY5Y cells were replaced with neurobasal media containing GlutaMAX and Opti-MEM, respectively. For TCA-precipitation, collected CM was centrifuged at 6000*g* for 5 min at 4 °C to remove cells and cell debris, and then one-fourth volume of 100% TCA was added to CM (43). CM were kept on ice for 30 min and centrifuged at 20,000*g* for 30 min at 4 °C. The resultant pellets were washed three times with cold acetone (Wako), air dried, and dissolved in 100 μl of Laemmli’s sample buffer with brief sonication.

### Preparation of EV-enriched fractions

We fractionated the CM of primary cortical neurons with step-wise centrifugation at 4 °C (24). Collected CM was centrifuged at 200*g* for 10 min and 2000*g* for 10 min to remove cellular debris and apoptotic bodies. The supernatants were centrifuged at 10,000*g* for 30 min using the TLA 100.3 rotor (Optima MAX-XP Ultracentrifuge, Beckman Coulter). The pellets were washed with 1 ml of phosphate-buffered saline (PBS; 10 mM phosphate, 137 mM NaCl, 2.7 mM KCl), suspended in PBS, and re-centrifuged at 10,000*g* for 30 min. Post-10,000*g* pellets were collected as large EV fractions. The resultant supernatants were centrifuged at 110,000*g* for 70 min. Post-110,000*g* supernatants were collected as non-EV fractions. The pellets were washed with 1 ml of PBS, suspended in PBS, and re-centrifuged at 110,000*g* for 70 min. The pellets were collected as small EV fractions. The supernatants of non-EV fractions were treated with TCA-precipitation. The pellets of large and small EV fractions and the non-EV fraction pellets precipitated with TCA were dissolved in the equal volume of Laemmli’s sample buffer. Samples were stored at −80 °C until use.

### Western blotting

Samples were denatured by boiling at 95 °C for 5 min in Laemmli’s sample buffer containing 2.5% 2-ME. Equal amounts of samples were applied to 13.5% polyacrylamide gels, and gels were transferred to PVDF membranes (0.45 μm pore size, Immobilon-P, Merck Millipore). To prevent detachment of proteins from membranes, transferred membranes were incubated in PBS containing 4% paraformaldehyde (PFA) and 0.01% glutaraldehyde (Electron Microscopy Sciences) for 30 min at room temperature (RT) (44). The membranes were washed in Tris-buffered saline (TBS; 25 mM Tris-HCl, pH 7.4, 137 mM NaCl, and 2.7 mM KCl) containing 0.05% (v/v) Tween 20 (TBS-T) for 5 min three times. After blocking membranes with 5% skim milk (BD Transduction Laboratories)/TBS-T for 30 min, membranes were probed with primary antibodies overnight at 4 °C followed by the corresponding horseradish peroxidase-conjugated secondary antibodies overnight at 4 °C. ECL plus and ECL (Thermo Fisher Scientific) were used for detection of proteins in CM and proteins in cell lysates, respectively. Signals were visualized by a CCD camera, Fusion FX7 (Vilbert Lourmat) or Amersham Imager 680 (GE Healthcare Life Sciences). Signal intensities were analyzed with a Quantity One software (Bio-Rad). The following primary antibodies were used: anti-α-syn (mouse monoclonal, 1:2500, 42/α-Synuclein, BD Transduction Laboratories), anti-β-actin (mouse monoclonal, 1:10,000, AC-15, Sigma-Aldrich), anti-LC3 (mouse monoclonal, 1:2000, 8E10, MBL), anti-p62 C-terminal (guinea pig polyclonal, 1:2000, PM066, MBL), anti-p62 (rabbit polyclonal, 1:200, #5114, Cell Signaling Technology), anti-Atg5 (mouse monoclonal, 1:1000, 4D3, MBL), anti-beclin 1 (rabbit monoclonal, 1:2500, EPR19662, Abcam), anti-phospho-AMPKα (Thr172) (rabbit monoclonal, 1:1000, 40H9, Cell Signaling Technology), anti-phospho-p70S6K (Thr389) (rabbit monoclonal, 1:1000, 108D2, Cell Signaling Technology), anti-Rab7 (rabbit monoclonal, 1:5000, EPR7589, Abcam), and anti-Rab8a (rabbit monoclonal, 1:5000, EPR 14873, Abcam), anti-FLAG M2 (mouse monoclonal, 1:1000, F1804, Sigma-Aldrich), anti-CD63 (rabbit monoclonal, 1:1000, EPR21151, Abcam), anti-amyloid-β precursor protein (APP) (mouse monoclonal, 1:1000, 22C11, Thermo Fisher), anti-flottilin-1 (mouse monoclonal, 1:1000, 18/Flotillin-1, BD Transduction Laboratories), anti-tau (rat monoclonal, 1:1000, RTM38, Wako), anti-SOD1 (rabbit polyclonal, 1:1000, GTX100554, GeneTex), anti-ubiquitin B (rabbit polyclonal, 1:1000, 10201-2-AP, Proteintech) antibodies. To detect p62 protein on the blots, anti-p62 antibody (PM066) was used, unless otherwise stated.

### Immunocytochemistry

Primary cortical neurons were plated at a density of 1.0 × 10^5^ cells per well on PLL-coated 4-chamber slides (Thermo Fisher Scientific). Neurons were incubated with artificial cerebrospinal fluid containing PBS or 10 μM glutamate for 30 min, pre-permeabilized in 0.1% digitonin (Fujifilm)/PBS for 10 s, fixed in 4% PFA for 15 min, and incubated with 0.2% Triton X-100/PBS for 5 min at RT. After blocking with 5% skim milk, they were labeled with a mixture of primary antibodies overnight at 4 °C and incubated with a mixture of Alexa Fluor 488 donkey anti-mouse IgG (H + L) (1:500, Jackson ImmunoResearch) and Alexa Fluor 594 donkey anti-rabbit IgG (H + L) (1:500) at RT for 2 h. Then, DAPI (1:500, Nacalai tesque, Inc) was added for 1 h at RT. Slides were analyzed with a laser-scanning confocal microscope (TCS SP8, Leica). For analysis of wt-αS/SH cells transfected with AcGFP1-LC3 cDNA, cells were plated at a density of 1.0 × 10^5^ cells per well on Geltrex-coated 4-chamber slides (Thermo Fisher Scientific), and Alexa Fluor 488 donkey anti-mouse IgG (H + L) and Alexa Fluor 594 goat anti-guinea pig IgG (H + L) (1:500, Thermo Fisher Scientific) were used as secondary antibodies. The following primary antibodies were used: anti-α-syn (mouse monoclonal, 1:100, 42/α-Synuclein), anti-α-syn (rabbit polyclonal, 1:1000, ab6176, Abcam), anti-LC3 (mouse monoclonal, 1:50, 4E12, MBL), anti-p62 C-terminal (guinea pig polyclonal, 1:500, PM066), and anti-LAMP1 (rabbit monoclonal, 1:100, EPR21026, Abcam) antibodies.

### Immunoprecipitation

The pellets prepared from TCA-precipitated CM were dissolved in ice-cold RIPA buffer (20 mM Tris-HCl, pH 7.4, 150 mM NaCl, 1% Triton X-100, 0.1% SDS, 0.5% deoxycholic acid, 10% glycerol, 1× protease inhibitor mixture, 1 mM EDTA, 5 mM NaF, 1 mM Na_3_VO_4_, 1× phosSTOP), and kept on ice for 30 min. After centrifugation at 12,000*g* for 30 min at 4 °C, the resultant supernatants were collected. The supernatants were incubated with anti-FLAG M2 antibody overnight at 4 °C, and then incubated with Protein G-agarose beads (GE Healthcare) for 2 h. Beads were washed three times with ice-cold RIPA buffer, and the immunoprecipitants were dissolved from beads by heating in Laemmli’s sample buffer. Equivalent amounts of samples were analyzed by Western blotting.

### Statistical analysis

Statistical analysis was performed using SPSS software (version 17.0; IBM Corp.). Comparison of two groups was performed using unpaired Student’s *t* test. Comparisons of three or more groups were performed using one-way analysis of variance (ANOVA) followed by Bonferroni’s *post hoc* tests, when the data showed homogeneity of variance according to Levene’s test. For inhomogeneous data, comparisons were performed using Welch’s ANOVA with Games-Howell’s *post hoc* tests. Data are expressed as mean ± standard deviation (SD), and *p* values <0.05 were considered statistically significant.

## Data availability

All data are contained within the article.

## Supporting information

This article contains supporting information.

## Conflict of interest

The authors declare that they have no known competing financial interests or personal relationships that could have appeared to influence the work reported in this paper.
