# Supplementary material for: Neuronal activity promotes secretory autophagy for the extracellular release of α-synuclein
Source: J Biol Chem. 2024 May 28;300(7):107419. doi: 10.1016/j.jbc.2024.107419 (PMC11253543; doi:10.1016/j.jbc.2024.107419)
Supplement: Supporting Figures S1–S3 [file mmc1.pdf]

Supporting information

Neuronal activity promotes secretory autophagy for the extracellular release of α-synuclein

Yoshitsugu Nakamura[1†], Taiki Sawai[1†], Kensuke Kakiuchi[1] and Shigeki Arawaka[1*]

[1]Department of Internal Medicine IV, Division of Neurology, Osaka Medical and Pharmaceutical University Faculty of Medicine. 2-7 Daigaku-machi, Takatsuki, Osaka 569-8686, Japan

[*]Address correspondence to Shigeki Arawaka, Department of Internal Medicine IV, Division of Neurology, Osaka Medical and Pharmaceutical University Faculty of Medicine. 2-7 Daigaku-machi, Takatsuki, Osaka 569-8686, Japan
Tel: +81-72-683-1221; FAX: +81-72-684-7087; E-mail: shigeki.arawaka@ompu.ac.jp.

Contents:

1. Figure S1: Characterization of anti-p62 antibodies.
2. Figure S2: Assessments of the secreted and intracellular levels of target molecules.
3. Figure S3: Effects of chloroquine on co-localization of LC3 puncta and p62 in SH-SY5Y cells.

Supplementary figure 1

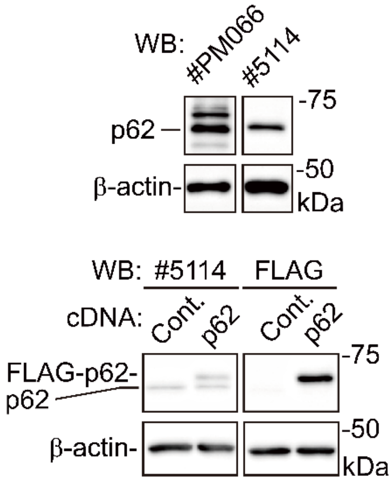

Figure S1. Characterization of anti-p62 antibodies.

Upper panels show western blots of primary cortical neuron lysates with two anti-p62 antibodies (#PM066 and #5114). These strips were cropped from the same blot. Lower panels show western blots of cell lysates from SH-SY5Y cells transfected with FLAG-p62 cDNA with anti-p62 (#5114) and anti-FLAG antibodies. These strips were cropped from the same blot. Cont., control; WB, western blot.

Supplementary figure 2

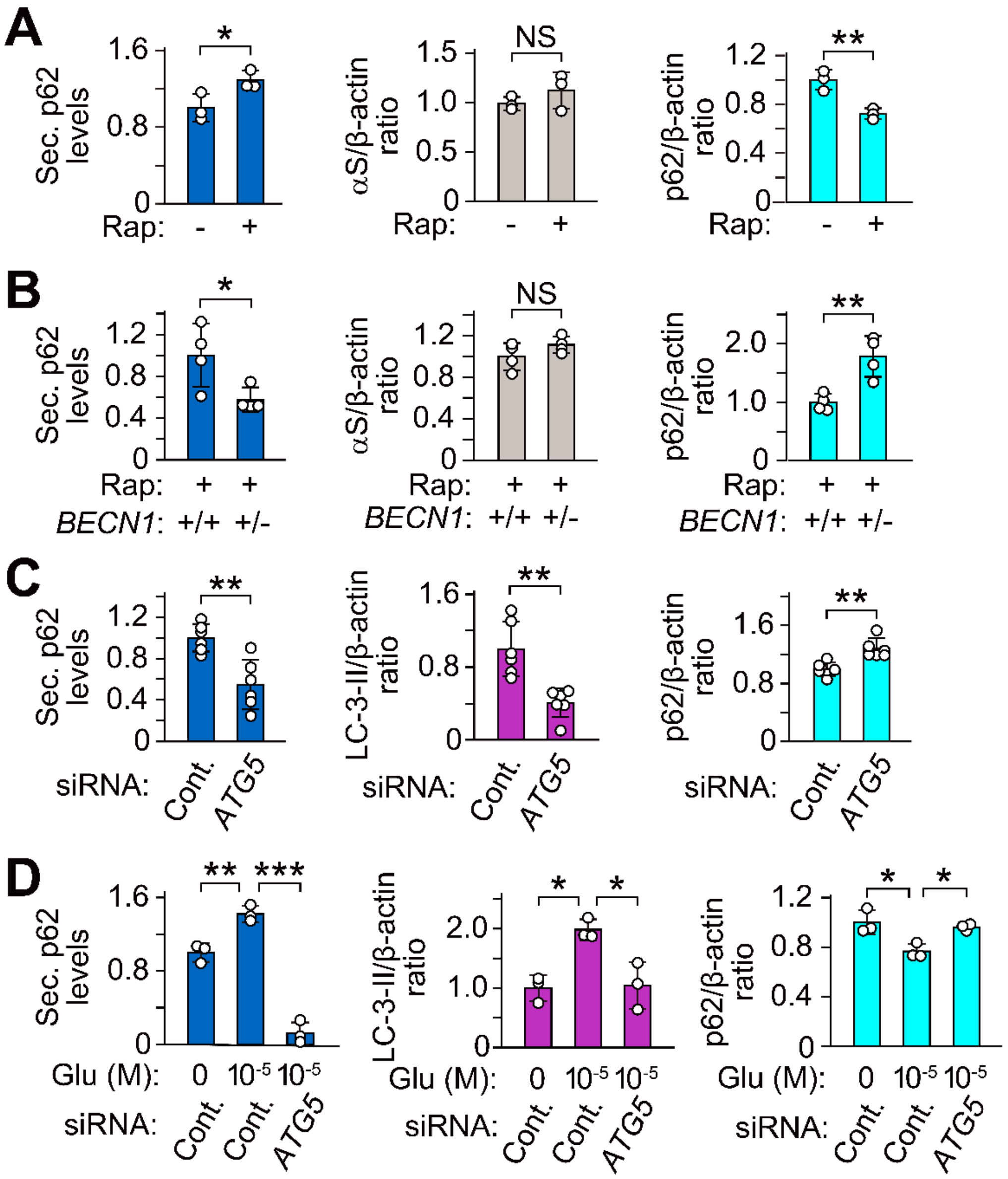

Figure S2. Assessments of the secreted and intracellular levels of target molecules.

These graphs show the remaining quantitative analysis of the blot data in Figure 2. *A*, comparisons of secreted p62 levels (left, blue columns), intracellular α-syn levels (middle, gray columns), and intracellular p62 levels (right, cyan columns) between primary cortical neurons treated with and

without rapamycin (n=3). Primary cortical neurons were originated from wild-type littermates of *BECN1*$^{+/-}$ mice. *B*, comparisons of secreted p62 levels (left, blue columns), intracellular α-syn levels (middle, gray columns), and intracellular p62 levels (right, cyan columns) between primary cortical neurons from wild-type littermates and *BECN1*$^{+/-}$ mice (n=4). These neurons were treated with rapamycin. *C*, comparisons of secreted p62 levels (left, blue columns), intracellular LC3-II levels (middle, purple columns), and intracellular p62 levels (right, cyan columns) between wt-αS/SH cells transfected with non-silencing control or *ATG5* siRNA oligonucleotides (n=6). *D*, comparisons of secreted p62 levels (left, blue columns), intracellular LC3-II levels (middle, purple columns), and intracellular p62 levels (right, cyan columns) among wt-αS/SH cells transfected with non-silencing control or *ATG5* siRNA oligonucleotides in the absence and presence of glutamate (secreted p62: n=3; $F_{(2,6)}$=114.577, p < 0.001, ANOVA); (LC3-II: n=3; $F_{(2,6)}$=11.913, p = 0.008, ANOVA); (intracellular p62: n=3; $F_{(2,6)}$=9.977, p = 0.012, ANOVA). Data represent mean ± SD. Data are analyzed by unpaired Student's *t* test (*A-C*) and one-way ANOVA with Bonferroni's *post hoc* tests (*D*). **p* < 0.05, ***p* < 0.01, ****p* < 0.001. Cont., control; Glu, glutamate; NS, not significant; Rap, rapamycin; Sec. p62, secreted p62; αS, α-synuclein.

Supplementary figure 3

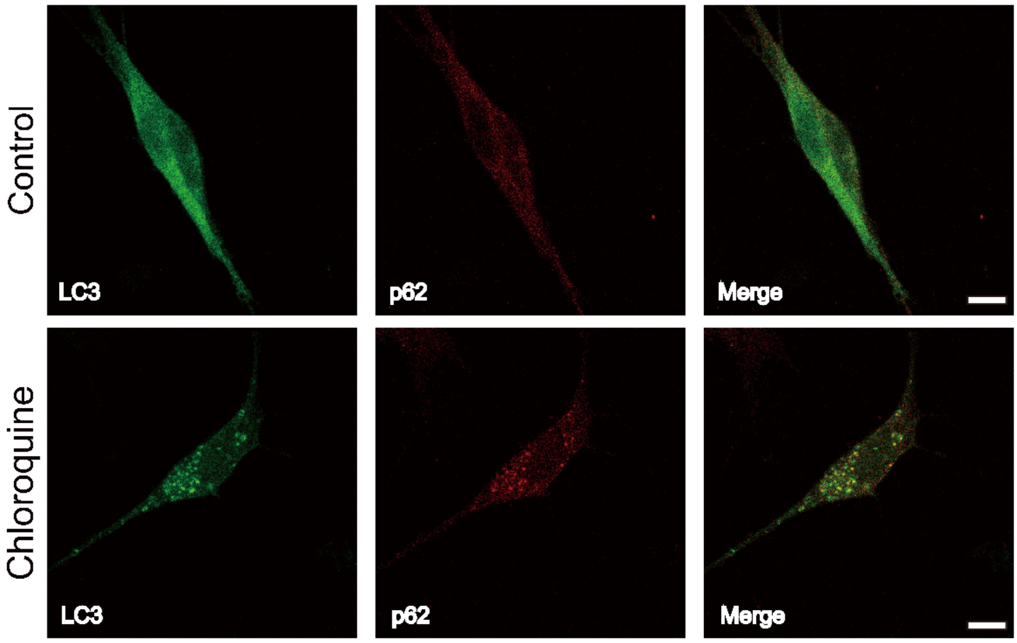

Figure S3. Effect of chloroquine on co-localization of GFP-LC3 puncta and p62 in SH-SY5Y cells.

Immunofluorescent analysis of SH-SY5Y cells co-treated with chloroquine and glutamate. The cells were transfected with AcGFP1-LC3 cDNA. Then, they were treated with 50 µM chloroquine for 6 h, followed by co-treatment with 10 µM glutamate for 30 min. Micrographs show images stained with anti-LC3 (4E12, green, left panels) and anti-p62 C-terminal (PM066, red, middle panels). Right micrographs show merged images. Scale bar: 8 µm.
